# Ultrasound-Assisted Extraction of Total Phenolic Compounds from *Inula helenium*

**DOI:** 10.1155/2013/157527

**Published:** 2013-08-19

**Authors:** Jin Wang, Yong-Ming Zhao, Ya-Ting Tian, Chun-Lin Yan, Chun-Yan Guo

**Affiliations:** ^1^Department of Pharmacy, Hebei North University, Zhangjiakou, Hebei 075000, China; ^2^Basic Medicine College, Hebei North University, Zhangjiakou, Hebei 075000, China

## Abstract

Ultrasound-assisted extraction (UAE) of phenolic compounds from *Inula helenium* was studied. Effects of ethanol concentration, ultrasonic time, solid-liquid ratio, and number of extractions were investigated. An orthogonal array was constructed to optimize UAE process. The optimized extraction conditions were as follows: ethanol concentration, 30%; solid-liquid ratio, 1 : 20; number of extractions, 2 times; extraction time, 30 min. Under the optimal conditions, the yield of total phenolic compounds and chlorogenic acid was 6.13 ± 0.58 and 1.32 ± 0.17 mg/g, respectively. The results showed that high amounts of phenolic compounds can be extracted from *I. helenium* by ultrasound-assisted extraction technology.

## 1. Introduction

*Inula helenium* L.(Compositae), also known as elecampane, is commonly found in the north of China. In traditional medicine, it is extensively used primarily for treatment of abdominal pain, emesis, diarrhea, and threatened abortion [1]. In addition, the roots are also listed in some European pharmacopoeias as a diuretic, diaphoretic, expectorant, and anthelmintic remedy [2]. Previous researches have shown evidence that *I. helenium* contains sesquiterpene lactones and flavonoids [3–7], while many studies have reported that a large amount of phenolic compounds present in the Compositae [8, 9]. However, no relevant work on total phenolic compounds (TPC) from *I. helenium* has been reported in the literature. 

Phenolic compounds are mainly distributed in the plant kingdom. Due to the special chemical structure, many phenolic compounds have antioxidant and free radical scavenging properties. Recent studies have indicated that phenolic compounds have antioxidant [10], antiviral [11], anti-inflammatory [12], antitumor [13], and immunomodulatory effects [14]. Accordingly, the extraction of phenolic compounds from natural products has become a hotspot. 

Extraction of phenolic compounds from medicinal plants can be carried out in various ways, such as Soxhlet, maceration, heat reflux, and microwave-assisted extraction. Although these techniques have been used for many years, it is important to note that these extraction steps could cause the loss of active ingredients, because of the hydrolysis, oxidation, and thermal decomposition during the high temperature extraction [15]. 

Ultrasound-assisted extraction (UAE) has proved to be a particularly effective extraction method to reduce the extraction temperature and amount of solvent and shorten the extraction time, which is especially useful for the extraction of thermosensitive and unstable compounds. Therefore, UAE has been widely used in the literature for the extraction of biologically active compounds, including the extraction of geniposide from *Gardenia jasminoides* [16], extraction of syringin from *Ilex rotunda* [17].

In this study, a method for ultrasound-assisted extraction (UAE) of TPC from *I. helenium* was investigated, and the operational parameters were optimized using orthogonal test. 

## 2. Results and Discussion 

### 2.1. Effect of Extraction Solvent on Yield of TPC and Chlorogenic Acid

**Figure 1:**
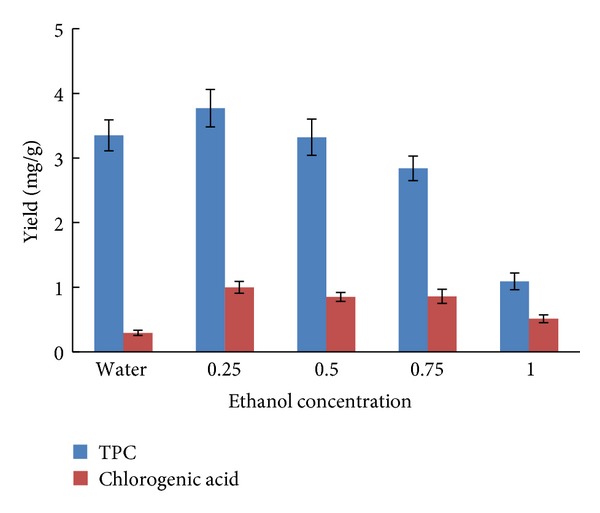
The HPLC chromatogram of chlorogenic acid (1) in the root of *I. helenium *(Column, Hypersil C_18_ column (200 mm × 4.6 mm); mobile phase, acetonitrile: 0.1% phosphoric acid (10 : 90); flow rate, 1 mL/min; column temperature, 30°C; wavelength, 327 nm; injection volume, 20 *μ*L).

The choice of solvents depends on the chemical properties of the components which would be extracted from a matrix. It was important in the extraction of TPC and chlorogenic acid, so water and different ethanol concentrations were chosen to evaluate the role of the extraction solvent. The yield of TPC and chlorogenic acid, as Figure 1 showed, reached a maximum when the ethanol concentration was 25%. The same phenomenon was also found in other experiments [18, 19]. Hence, subsequent experiments were conducted with 25% ethanol.

### 2.2. Effect of Solid-Liquid Ratio on Yield of TPC and Chlorogenic Acid

**Figure 2:**
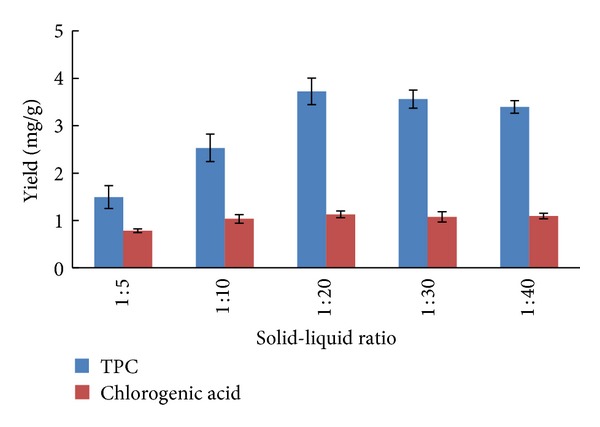
Effect of extraction solvent on TPC and chlorogenic acid yield (solid-liquid ratio, 1 : 15; extraction time, 30 min; number of extractions, 1; *n* = 5).

To determine the effect of solid-liquid ratio on extraction yield, experiments were carried out at ratio ranging between 1 : 5 and 1 : 40. The UAE experiments were set as follows: 25% ethanol solvent and extraction time of 30 min. As shown in Figure 2, the extraction yield was initially increased when the ratio increased from 1 : 5 to 1 : 20 and then remained fairly constant. Therefore, the solid-liquid ratio of 1 : 20 was chosen for further optimization studies.

### 2.3. Effect of Ultrasonic Time on Yield of TPC and Chlorogenic Acid

**Figure 3:**
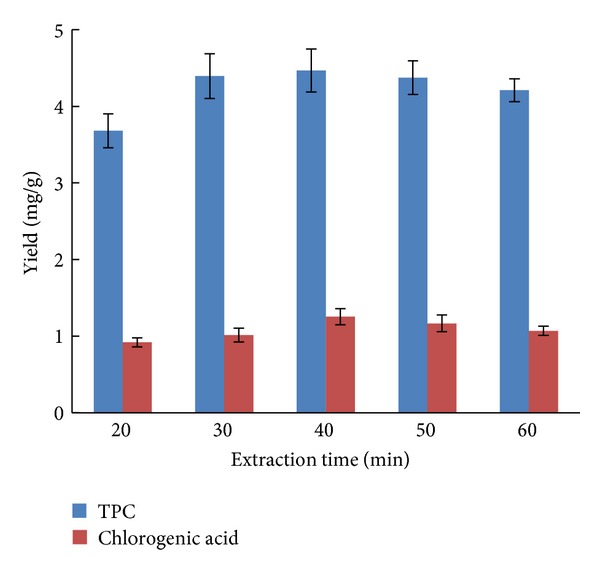
Effect of solid-liquid ratio on TPC and chlorogenic acid yield (ethanol concentration, 25%; extraction time, 30 min; number of extractions, 1; *n* = 5).

In order to obtain the maximum yield of TPC and chlorogenic acid from the root of *I. helenium*, ultrasound-assisted extractions were performed at five extraction time (20, 30, 40, 50 and 60 min). The effect of different extraction time on yield of TPC and chlorogenic acid is shown in Figure 3. It was reported that long period of extraction time favors the phenolic compounds production [20]. Likewise, at constant ethanol concentration and solid-liquid ratio, increasing the extraction time significantly increased the yield at the initial stage. But further increased in the ultrasonic time did not show any increase in the total phenolic content when the extraction was more than 40 min. Accordingly, 40 min was chosen as the extraction time in succeeding experiments.

### 2.4. Effect of Number of Extractions on Yield of TPC and Chlorogenic Acid

**Figure 4:**
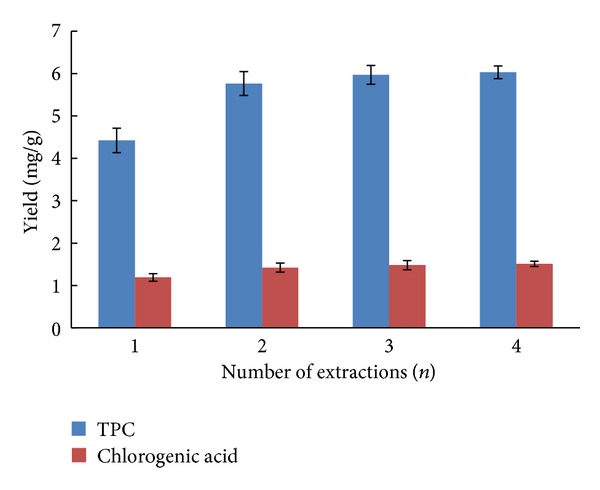
Effect of ultrasonic time on TPC and chlorogenic acid yield (ethanol concentration, 25%; solid-liquid ratio, 1 : 20; number of extractions, 1; *n* = 5).

In order to evaluate the number of extractions on yield of TPC and chlorogenic acid, four different numbers of extractions were applied to the extraction experiments, respectively. It can be seen from Figure 4 that the yield of TPC and chlorogenic acid was first increased with increasing extraction times, and a relatively high yield of TPC and chlorogenic acid was achieved when the samples were extracted for 3 times. 

### 2.5. Orthogonal Design Experiment

 An orthogonal array of four factors and three levels was constructed to optimize UAE conditions. The experimental design and data analysis are shown in Table 1. The *Km* (*m* = 1–3) values are the averages of TPC or chlorogenic acid of every factor at each level. *R* value is the range of *K* value. According to the *R* value, it can be observed that there were great differences between each factor. The number of extractions was found to be the most important factor, afterward followed by ethanol concentration, ultrasonic time, and solid-liquid ratio. Therefore the maximum yield of TPC and chlorogenic acid was obtained when the conditions were *C*_2_ *A*_3_ *D*_2_ *B*_2_, namely, number of extractions 2 times, 30% ethanol as the solvent, ultrasonic time of 40 min, and solid-liquid ratio of 1 : 20, respectively. Through confirmatory test, the yield of TPC and chlorogenic acid was 6.13 ± 0.58 and 1.32 ± 0.17 mg/g, respectively. The result indicated that the extraction efficiency was superior to each group in the orthogonal experiment.

## 3. Experimental Section 

### 3.1. Plant Materials and Chemical

Dried roots of *I. helenium* were purchased from a herbal medicine market (Anguo, China) and identified by Professor Heng-cheng Zhao (College of Traditional Chinese Medicine, Hebei North University). The specimen (no. 2012-11) was kept in the Department of Pharmacy, HeBei North University. The root was pulverized and sifted through a 60-mesh sieve. Folin-Ciocalteu reagent and gallic acid were purchased from Sigma Chemical Co. Acetonitrile (HPLC grade) was obtained from Adamas (Shanghai, China). Ethanol was analytical grade and used for the extraction of phenolic compounds.

### 3.2. Ultrasound-Assisted Extraction Process

UAE was performed using an ultrasonic cleaning bath (KQ-250V, Kun-Shan Ultrasonic Instruments Co., Ltd, Kunshan, China). The extraction variables were set as follows: ethanol solutions 0, 25, 50, 75, and 100%, solid-liquid ratios 1 : 5, 1 : 10, 1 : 20, 1 : 30, and 1 : 40, time of sonication 20, 30, 40, 50, and 60 min. Ultrasound equipment operated at a frequency of 40 KHz, 100 W of power, and temperature of 25°C. Dried powder of *I. helenium* (5.0 g) was mixed with solvent in a 250 mL conical flask. The flask was immersed into the ultrasonic bath and extracted at different conditions. After extraction, the extract was centrifuged for 15 min at 3000 rpm for deposit suspension particle and utilized for further analysis. 

### 3.3. Experimental Design and Data Analysis

Based on preliminary experiments, an orthogonal L_9_(3)^4^ test design was used to optimize UAE conditions. The orthogonal experiment was carried out with four factors and three levels, namely, ethanol concentration (20%, 25%, and 30%), solid-liquid ratios (1 : 15, 1 : 20, and 1 : 25 g/mL), number of extractions (1, 2, and 3), and ultrasonic times (35, 40, and 45 min). The factors and levels for orthogonal test are displayed in Table 2. All the experiments were performed in triplicate, and the data were expressed as the mean ± SD (standard deviation). 

### 3.4. Determination of TPC

The total phenolic contents in the extracts were measured by using the Folin-Ciocalteu method [21]. In brief, the diluted extracts solution (0.5 mL) was mixed with Folin-Ciocalteu reagent (0.5 mL) and saturated sodium carbonate solution (10 mL). The mixture was then diluted to 25 mL with distilled water and allowed to stand at room temperature for 30 min. The absorbance of the solution was measured at 760 nm using a UV-VIS spectrophotometer (model 2100, Labtech, USA). The total phenolic content was expressed as gallic acid equivalents in milligrams per gram of sample. The determination of phenolic compounds in the extracts was performed in triplicate, and the results were averaged.

### 3.5. Determination of Chlorogenic Acid

**Figure 5:**
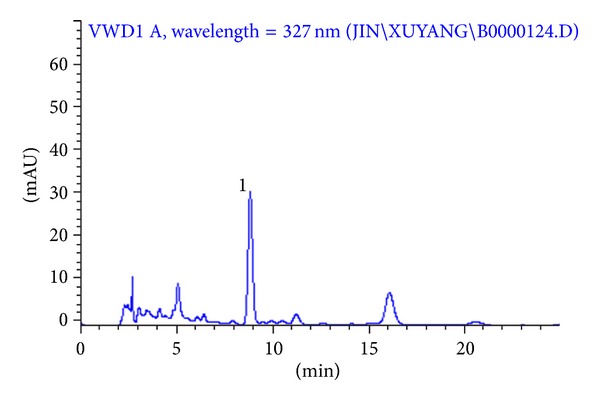
Effect of number of extraction on TPC and chlorogenic acid yield (ethanol concentration, 25%; solid-liquid ratio, 1 : 20; extraction time, 40 min; *n* = 5).

The chlorogenic acid in *I. helenium* was quantified by high performance liquid chromatography (HPLC) with UV detection. Quantitative HPLC was performed on an Agilent HP 1100 series HPLC system (Agilent Technologies, USA) consisting of a quaternary pump solvent delivery system, an autodegasser, a column oven, and UV detector. Separations were performed on a Hypersil C_18_ column (200 mm × 4.6 mm, i.d. 5 *μ*m particle size). The mobile phase was acetonitrile: 0.1% phosphoric acid (10 : 90) with a flow rate of mL/min. The detection wavelength and column temperature were set at 327 nm and 30°C, respectively. The injection volume was 20 *μ*L. The HPLC chromatogram of chlorogenic acid in the root of *I. helenium* is shown in Figure 5. 

## 4. Conclusions 

In the present study, an optimized ultrasound-assisted extraction method of total phenolic compounds from* I. helenium* has been developed. This is the first report on the extraction of phenolic compounds from* I. helenium. *The results of this study showed that UAE was a suitable and economical method for the extraction of total phenolic compounds. 

## Figures and Tables

**Table 1:** Orthogonal design and experimental results.

No.	Ethanol concentration	Solid-liquid ratio	Number of extractions	Ultrasonic time	TPC (mg/g)	Chlorogenic acid (mg/g)
1	1	1	1	1	3.48 ± 0.38	0.89 ± 0.07
2	1	2	2	2	5.81 ± 0.44	1.36 ± 0.12
3	1	3	3	3	4.47 ± 0.41	1.02 ± 0.11
4	2	1	2	3	5.01 ± 0.46	1.19 ± 0.14
5	2	2	3	1	4.31 ± 0.41	0.98 ± 0.12
6	2	3	1	2	3.53 ± 0.37	0.86 ± 0.06
7	3	1	3	2	5.21 ± 0.47	1.29 ± 0.13
8	3	2	1	3	4.23 ± 0.39	1.13 ± 0.09
9	3	3	2	1	5.57 ± 0.52	1.39 ± 0.12
*K*1*t *	4.59	4.56	3.75	4.45		
*K*2*t *	4.28	4.78	5.46	4.85		
*K*3*t *	5.00	4.52	4.46	4.57		
*Rt*	0.72	0.26	1.71	0.39		
*K*1*c*	1.09	1.12	0.96	1.09		
*K*2*c*	1.01	1.16	1.31	1.17		
*K*3*c*	1.27	1.09	1.10	1.11		
*Rc*	0.26	0.07	0.35	0.08		

*t*: total phenolic compounds; *c*: chlorogenic acid.

**Table 2:** Factors and levels of the orthogonal design.

Influence factors	Level		
	1	2	3
(A) Ethanol concentration	20%	25%	30%
(B) Solid-liquid ratio/g/mL	1 : 15	1 : 20	1 : 25
(C) Number of extractions	1	2	3
(D) Ultrasonic time	35 min	40 min	45 min
